# A Novel Transcript Isoform of TBK1 Negatively Regulates Type I IFN Production by Promoting Proteasomal Degradation of TBK1 and Lysosomal Degradation of IRF3

**DOI:** 10.3389/fimmu.2020.580864

**Published:** 2020-09-30

**Authors:** Jie Zhang, Xiao Man Wu, Yi Wei Hu, Ming Xian Chang

**Affiliations:** ^1^State Key Laboratory of Freshwater Ecology and Biotechnology, Key Laboratory of Aquaculture Disease Control, Institute of Hydrobiology, Chinese Academy of Sciences, Wuhan, China; ^2^University of the Chinese Academy of Sciences, Beijing, China; ^3^Innovation Academy for Seed Design, Chinese Academy of Sciences, Beijing, China

**Keywords:** TBK1 isoform, TBK1, IRF3, ubiquitination, protein degradation

## Abstract

TANK-binding kinase 1 (TBK1), an IKK-related serine/threonine kinase, is pivotal for the induction of antiviral type I interferon (IFN) by TLR and RLR signaling pathways. In a previous study, we demonstrated that TBK1 spliced isoforms (TBK1_tv1 and TBK1_tv2) from zebrafish were dominant negative regulators in the RLR antiviral pathway by targeting the functional TBK1–IRF3 complex formation. In this study, we show that the third TBK1 isoform (namely TBK1_tv3) inhibits zebrafish type I IFN production by promoting TBK1 and IRF3 degradation. First, ectopic expression of TBK1_tv3 suppresses poly(I:C)- and Spring viremia of carp virus-induced type I IFN response, and also inhibits the up-regulation of IFN promoter activities stimulated by RIG-I, MDA5, MAVS, TBK1, and IRF3. Second, TBK1_tv3 targets TBK1 and IRF3 to impair the formation of TBK1 dimer, TBK1–IRF3 complex, and IRF3 dimer. Notably, TBK1_tv3 promotes the degradation of TBK1 through the ubiquitin–proteasome pathway and the degradation of IRF3 through the lysosomal pathway. Further analysis demonstrates that TBK1_tv3 promotes the degradation of TBK1 for K48-linked ubiquitination by targeting the K251, K256, and K271 sites of TBK1. Collectively, our results suggest a novel TBK1 isoform-mediated negative regulation mechanism, which serves to balance the production of type I IFN and ISGs.

## Highlights

- TBK1_tv3 facilitates SVCV replication.
- TBK1_tv3 impairs the formation of TBK1/IRF3 homo- and hetero-dimerization.
- TBK1_tv3 promotes TBK1 degradation via the ubiquitin–proteasome pathway.
- TBK1_tv3 promotes IRF3 degradation via the lysosomal pathway.

## Introduction

Type I interferon (IFN) plays a vital role in inducing cell-intrinsic antimicrobial states in infected and neighboring cells to limit the spread of viral pathogens, modulating innate immune response in a balanced manner and activating the adaptive immune system to promote the development of T- and B-cell responses (1). However, aberrant production of type I IFN is harmful for the host, which can contribute to autoimmune diseases such as systemic sclerosis (2, 3). TANK-binding kinase 1 (TBK1) is a key kinase in type I IFN production and can be activated through a variety of immune signaling, for example, by double stranded (ds)-RNA via TLR3–TRIF pathway, by LPS via TLR4–TRIF pathway, by viral RNA via RIG-I–MAVS pathway, and by dsDNA via cGAS–STING pathway (4, 5). Activated TBK1 then phosphorylates IRF3 and induces the production of type I IFN (6, 7). Therefore, the regulation of TBK1 activity is an important mechanism for regulating antiviral response or maintaining immune homeostasis.

The structure of TBK1 consists of an N-terminal serine/threonine kinase domain (KD), a middle ubiquitin-like domain (ULD), and two C-terminal coiled-coil domains (namely CCD1 and CCD2). CCD1 is also referred to as a scaffold dimerization domain (SDD). TBK1 is regulated by adaptor proteins and many other molecules, which control its activation and participation in different signaling pathways. In mammals, multiple mechanisms including posttranslational modification of TBK1, kinase activity modulation, and prevention of functional TBK1-containing complexes formation are associated with the regulation of TBK1 activity (8, 9). For example, DYRK2 promotes proteasomal degradation of TBK1 to attenuate antiviral responses by phosphorylating TBK1 at Ser527 (10). The E3 ubiquitin ligase RNF128 activates TBK1 by promoting the K63-linked polyubiquitination of TBK1 at Lys30 and Lys401 (11). Many molecules such as TRIM26 and IFIT3 associate with TBK1 to facilitate the formation of TBK1-containing complexes; however, other molecules such as NLRC3 and NLRX1 prevent the interaction of TBK1 with other adaptors (12–15).

Both mammalian and piscine TBK1 undergo alternative splicing (16), and TBK1 isoforms play an inhibitory role in virus-triggered IFN signaling (17, 18). In terms of specific mechanism, the splice variant of mouse TBK1 lacking the N-terminal KD can bind to RIG-I through its coiled-coil domain and disrupt the interactions of RIG-I with VISA (17). Zebrafish TBK1 isoforms TBK1_tv1 and TBK1_tv2 can disrupt the formation of a functional TBK1–IRF3 complex and impede the phosphorylation of IRF3 mediated by TBK1 (18). Except TBK1_tv1 and TBK1_tv2, a weak protein band (∼65 kDa) was also identified in ZF4 cells with/without Spring viremia of carp virus (SVCV) infection, which may represent the TBK1_tv3 isoform in the present study. Compared with TBK1, TBK1_tv3 only lacks CCD2. It is unclear about the function of TBK1_tv3 and whether TBK1 activity can be regulated by its isoform(s) through ubiquitination pathway.

In this paper, we identified the sequence and functional characteristics of zebrafish TBK1_tv3 isoform. Unlike zebrafish TBK1_tv1 and TBK1_tv2, TBK1_tv3 contains the intact KD and ULD. Functionally, TBK1_tv3 negatively regulates fish IFN antiviral response by degrading TBK1 and IRF3 through the ubiquitin–proteasome and lysosomal pathway, respectively. Furthermore, the necessary ubiquitination sites of TBK1 targeted by TBK1_tv3 were identified. These findings shed light on a novel negative feedback regulation of TBK1 and IRF3 activities.

## Materials and Methods

### Ethics Statement

All animal experiments were conducted in accordance with the Guiding Principles for the Care and Use of Laboratory Animals and were approved by the Institute of Hydrobiology, Chinese Academy of Sciences (approval ID: IHB 2013724).

### Cell and Virus

Zebrafish ZF4 [embryonic fibroblast cell line, CRL-2050; American Type Culture Collection (ATCC)] was grown at 28°C in DMEM/F12 (1:1) medium supplemented with 10% FBS (Life Technologies), 100 U/ml penicillin, and 100 mg/ml streptomycin. EPC cells (epithelioma papulosum cyprinid cell line, CRL-2872; ATCC) were maintained in M199 supplemented with 10% FBS at 28°C. Human embryonic kidney 293T (HEK 293T) cells were obtained from the ATCC and cultured in DMEM supplemented with 10% FBS at 37°C. SVCV (ATCC: VR-1390) was propagated in EPC cells and stored at −80°C.

### Plasmid Construction and Transfection

Zebrafish plasmids pcDNA3.1-RIG-I, pcDNA3.1-MDA5, p3 × FLAG-MAVS (MAVS-FLAG), p3 × FLAG-TBK1 (TBK1-FLAG), pTurbo-TBK1-GFP (TBK1-GFP), pTurbo-IRF3-GFP (IRF3-GFP), p3 × FLAG-IRF3 (IRF3-FLAG), IFN1 (DrIFN1pro-luc), and IFN3 (DrIFN3pro-luc) were previously prepared and stored in our laboratory (18–21). TBK1_tv3-GFP and TBK1_tv3-FLAG were obtained using the primer pairs TBK1_tv3F/TBK1_tv3R, and cloned into the pTurboGFP-N (Everogen) and p3 × FLAG-CMV-14 (Sigma-Aldrich) vectors. TBK1 truncated and site mutants were generated by PCR and then cloned into p3 × FLAG-CMV-14 vector. The plasmids Ub-HA WT, Ub-HA K48, and Ub-HA K63 were obtained from Yossi Yarden (Weizmann Institute, Israel). Transfection of plasmids was performed in EPC cells and HEK 293T cells using Lipofectamine 2000 (Invitrogen). For all procedures, standard protocols were used according to the manufacturers’ manuals. The primers used for plasmid constructs are listed in Supplementary Table 1 in Supplementary Material.

### Antibodies and Reagents

Polyclonal antibodies (Abs) against zebrafish TBK1 were generated according to our previous method (18), which can recognize TBK1 normal form, TBK1_tv1 and TBK1_tv3 isoforms, but not for TBK1_tv2 isoform. Anti-FLAG and anti-HA mouse Abs, dimethylsulfoxide (DMSO), and FLAG Immunoprecipitation Kit were purchased from Sigma-Aldrich. The anti-pTurboGFP antibody was purchased from Everogen. The anti-GAPDH mouse antibody was purchased from Proteintech. Poly(I:C) was purchased from InvivoGen. The MG132 and NH_4_Cl were purchased from Selleck. Lipofectamine 2000 and protease inhibitor cocktail were purchased from Thermo Fisher Scientific.

### SVCV Immersion Infection in Zebrafish Larvae

To determine the role of zebrafish TBK1_tv3 in SVCV infection *in vivo*, p3 × FLAG or TBK1_tv3-FLAG were diluted to 200 ng/μl and microinjected into fertilized eggs at the one-cell stage. The hatched larvae (4 dpf) from each group were exposed to 2 × 10^6^ PFU/ml SVCV for 24 h, and then maintained in 60-mm sterile disposable Petri dishes with supplemental 25 ml fresh water. The number of surviving larvae was counted daily for 3 days. GraphPad Prism 7 was used to generate survival curves, and the log-rank test was used to test differences in survival with WT zebrafish microinjected with p3 × FLAG as the control group. In total, 8–10 larvae per group at 24 h post-infection (hpi) and 48 hpi were collected and used for Quantitative RT-PCR (qRT-PCR) to examine the expression of SVCV genes.

### Real-Time Quantitative RT-PCR

For inducible expression of TBK1_tv3 in response to SVCV infection, ZF4 cells were passaged in six-well plates at 1 × 10^6^ cells per well. After 24 h, these cells were infected with SVCV at a multiplicity of infection of 1, and then collected at 24, 48, and 72 hpi for RNA extraction using Trizol (Invitrogen). To determine the effect of TBK1_tv3 in regulating the expression of IFN1 and ISGs, TBK1-FLAG, TBK1_tv3-FLAG, or FLAG empty plasmid were diluted to the desired concentration of 100 ng/μl. These plasmids were microinjected into fertilized zebrafish eggs at one-cell stage, respectively. To determine the effect of TBK1_tv3 in regulating the expression of IFN1 and ISGs mediated by MAVS or TBK1, MAVS-FLAG, TBK1-FLAG, TBK1_tv3-FLAG, or FLAG empty plasmid was diluted to 100 ng/μl. Combination constructs of two plasmids were microinjected into fertilized zebrafish eggs at the one-cell stage, respectively. The typical injected volume was 2 nl. Injected embryos were raised at 28°C in fish water and collected at 24 and/or 48 hpf. For all experimental and control groups, 50 embryos per group were used for RNA extraction. qRT-PCR analysis was performed using an ABI Prism 7000 system using primers specific to individual genes (Supplementary Table 1 in Supplementary Material) or those described by others (21, 22). The relative expression of target genes was normalized to the expression of zebrafish GAPDH and expressed as fold changes relative to the corresponding control group.

### Luciferase Activity Assay

For luciferase activity assay, EPC cells seeded overnight in 24-well plates at 3 × 10^5^ cells per well were transiently transfected with various indicated plasmids with indicated DNA concentration, together with 25 ng Renilla (Promega), 250 ng DrIFN1pro-luc, or 250 ng DrIFN3pro-luc reporter plasmids. At 24 h post-transfection, the cells were transfected with poly(I:C) at a final concentration of 2 μg/ml or were left untreated. Another 24 h later, the cells were lysed. To examine the effect of TBK1_tv3 on the IFN induction mediated by RIG-I, MDA5, MAVS, TBK1, or IRF3, EPC cells seeded overnight were transiently transfected with various plasmids at the indicated DNA concentration. At 48 h post-transfection, the cells were lysed. Luciferase activity was measured using the Dual-Luciferase Reporter Assay System (Promega). Data were normalized to the Renilla luciferase activity and expressed as mean ± SEM of three independent experiments.

### Co-immunoprecipitation Assay and Western Blotting

To investigate the effect of TBK1_tv3 on the formation of TBK1 homodimers, IRF3 homodimers, or the formation of TBK1–IRF3 complex, EPC cells were transfected with the indicated plasmids. At 48 h post-transfection, the cells were washed with ice-cold PBS buffer and then lysed in IP lysis buffer containing Protease Inhibitor Cocktail. Co-immunoprecipitation (Co-IP) was performed using FLAG-Tagged Protein Immunoprecipitation Kit according to the manufacturer’s manual. The agarose was washed 8–10 times with ice-cold wash solution and protein was eluted with elution buffer. Total lysate and eluted proteins were analyzed by Western blotting (WB) using anti-GAPDH, anti-FLAG, and anti-pTurboGFP Abs.

To investigate the exact effect of TBK1_tv3 on the protein expression of TBK1, TBK1 mutants, or IRF3, EPC cells seeded overnight were transiently transfected with various plasmids at the indicated DNA concentration. At 48 h post-transfection, the cells were treated with MG132 or NH_4_Cl at the indicated concentration for another 6 h or left untreated. The cells were washed with PBS and then lysed in Pierce RIPA Buffer containing Protease Inhibitor Cocktail. The cell lysates were analyzed by WB with anti-GAPDH, anti-FLAG, and anti-pTurboGFP Abs.

### Ubiquitination Assays

TANK-binding kinase 1 or TBK1 mutant plasmid and Ub-HA WT, Ub-HA K63, or Ub-HA K48 plasmids were co-transfected into HEK 293T cells together with or without TBK1_tv3 plasmid. At 24 h post-transfection, cells were then treated with MG132 (20 μM) for 6 h. After this, the cells were washed with ice-cold PBS buffer and then lysed in IP lysis buffer containing Protease Inhibitor Cocktail. Ubiquitination assays were performed using FLAG-Tagged Protein Immunoprecipitation Kit according to the manufacturer’s manual. The agarose was washed four times with ice-cold wash solution and protein was eluted with elution buffer. Total lysate and eluted proteins were analyzed by WB using anti-GAPDH, anti-FLAG, anti-HA, and anti-pTurboGFP Abs.

### Statistical Analysis

Statistical analysis and graphs were performed and produced using GraphPad Prism 7.0 software. Data from qRT-PCR are presented as mean and SEM. The significance of results was analyzed by an ANOVA test or Student’s *t*-test (*p < 0.05, **p < 0.01).

## Results

### TBK1_tv3 Is a Novel Isoform of TBK1

**FIGURE 1:**
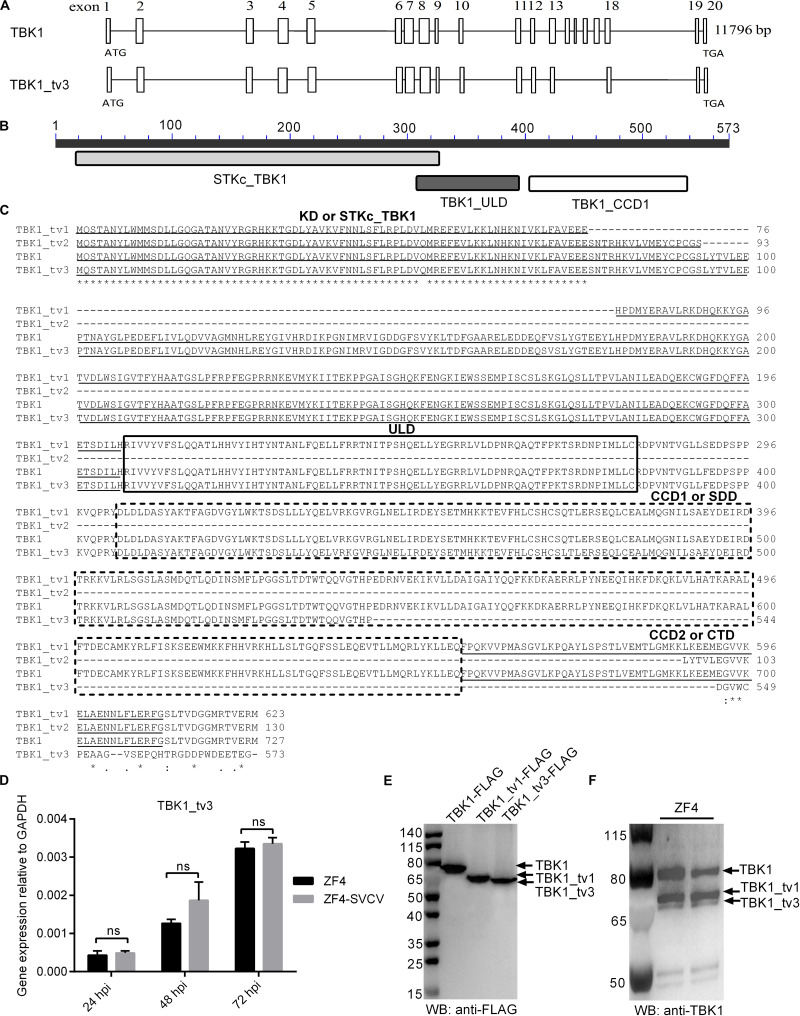
Characterization of zebrafish TBK1_tv3 isoform. **(A)** The alternative splicing of zebrafish TBK1_tv3. **(B)** Protein domains of zebrafish TBK1_tv3. **(C)** Multiple alignments of amino acid sequences of zebrafish TBK1 and its isoforms TBK1_tv1, TBK1_tv2, and TBK1_tv3. **(D)** The mRNA expression of zebrafish TBK1_tv3 in ZF4 cells with/without SVCV infection. Total RNA was extracted from ZF4 including 24, 48, and 72 h post-infection. Data represented means ± SEM (*n* = 3) and were tested for statistical significance. ns, not significant. **(E)** The exogenous expression of TBK1, TBK1_tv1, and TBK1_tv3 in EPC cells. **(F)** The endogenous expression of TBK1, TBK1_tv1, and TBK1_tv3 in ZF4 cells. For **E,F**, total protein was extracted at 24 h.

Besides the full-length form of TBK1, two truncated isoforms of TBK1 (zebrafish TBK1_tv1 and TBK1_tv2) are characterized in our previous study, which are generated by alternative splicing of different exons (18). In this study, the third transcript of zebrafish TBK1 was identified and cloned unintentionally, and was named as TBK1_tv3 (GenBank accession no. MT430746). By sequence aligning with TBK1 normal form, TBK1_tv3 lacks exons 14–17, 9 bp at the C terminal of exon 13, 34 bp at the N terminal of exon 18, and 17 bp in the middle of exon 19 (Figure 1A), which leads to an in-frame deletion of the C-terminal CCD2 domain of TBK1 (amino acids 658–713; Figures 1B,C). Different from zebrafish TBK1_tv1 and TBK1_tv2 (18), TBK1_tv3 is generated by exon skipping with the usage of the same ATG transcription start site and different stop codon site.

The mRNA and protein expressions of TBK1_tv3 were examined in ZF4 cells with or without SVCV infection. Although the obvious expression of SVCV-N in all infected samples, SVCV infection failed to induce the mRNA expression of TBK1_tv3 at 24, 48, and 72 hpi (Figure 1D). The sizes of TBK1, TBK1_tv1, and TBK1_tv3 were confirmed by WB in EPC cells transfected with TBK1-FLAG, TBK1_tv1-FLAG, or TBK1_tv3-FLAG. The size of TBK1_tv3 was very close to TBK1_tv1 (Figure 1E). The immunogen of zebrafish TBK1 Abs is specific for the CCD1 and CCD2 domains of TBK1. TBK1 and TBK1_tv1 contain the intact CCD1 and CCD2 domains, and a deletion of CCD2 domain for TBK1_tv3. Compared with TBK1 and TBK1_tv1, the endogenous expression of TBK1_tv3 protein was rather weak in ZF4 cells, which might be due to the poor recognition of zebrafish TBK1 Abs toward TBK1_tv3 antigen (Figure 1F).

### Negative Regulation of TBK1_tv3 on the IFN and ISGs

**FIGURE 2:**
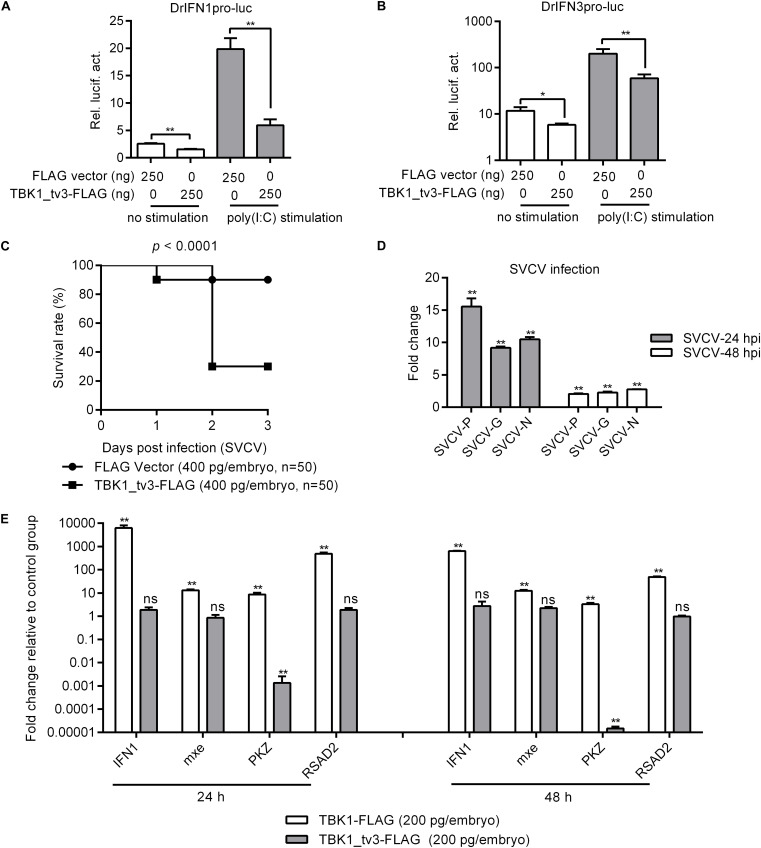
The effect of zebrafish TBK1_tv3 on the regulation of IFNs and ISGs. **(A)** Effect of zebrafish TBK1_tv3 on the IFN1 (DrIFN1pro-luc) promoter with/without poly(I:C) stimulation. **(B)** Effect of zebrafish TBK1_tv3 on the IFN3 (DrIFN3pro-luc) promoter with/without poly(I:C) stimulation. For **A,B**, EPC cells seeded in 24-well plates were co-transfected with 250 ng various indicated plasmids, together with 25 ng Renilla and 250 ng IFN1 or IFN3 reporter plasmids. 24 h post-transfection, the cells were stimulated with poly(I:C) or left untreated. Another 24 h later, the cells were harvested for the detection of luciferase activity. Data represented means ± SEM (*n* = 3) and were tested for statistical significance. *p < 0.05 and **p < 0.01. **(C)** The overexpression of zebrafish TBK1_tv3 decreased the survival rate of zebrafish larvae infected with SVCV. **(D)** The overexpression of zebrafish TBK1_tv3 increased the expression of SVCV genes. For **C,D**, zebrafish larvae microinjected with p3XFLAG or TBK1_tv3-FLAG were infected with 2 × 10^6^ PFU/ml SVCV for 24 h and monitored for 3 days. Samples were collected at 24 and 48 hpi, and used for qRT-PCR. **(E)** Effect of zebrafish TBK1 and TBK1_tv3 on expression of ISGs. Embryos were microinjected at the one-cell stage with 100 ng of indicated expression construct. At 24 and 48 h post-fertilization, 50 embryos for each sample were used for RNA extraction. The p3xFLAG-injected group was used for control. For all qRT-PCR, data represented the means ± the SEM (*n* = 3) and were tested for statistical significance. **p < 0.01; ns, not significant.

TANK-binding kinase 1 is the critical kinase involved in the induction of type I IFN in response to stimulation by pathogen-associated molecular patterns (8). To reveal the possible regulation of TBK1_tv3 in antiviral immunity, the IFN1 and IFN3 promoter activities were firstly investigated by dual-luciferase reporter assay. The overexpression of TBK1_tv3 markedly inhibited the activation of zebrafish IFN1 and IFN3 promoters with or without poly(I:C) stimulation (Figures 2A,B). Next, the role of TBK1_tv3 overexpression in the immune response of zebrafish larvae against SVCV infection was characterized. Notably, SVCV-infected zebrafish larvae microinjected with TBK1_tv3 exhibited reduced survival compared with zebrafish larvae microinjected with FLAG empty plasmid, with the elevated expression of *SVCV-N*, *SVCV-G*, and *SVCV-P* both at 24 and 48 hpi (Figures 2C,D). Last, the function of TBK1_tv3 in the regulation of IFN1 and ISGs was measured by qRT-PCR. Different from TBK1, the overexpression of TBK1_tv3 failed to induce the expression of IFN1 and ISGs, even significantly inhibited the expression of PKZ with the 734.75-fold decrease at 24 h and 67,652.59-fold decrease at 48 h (Figure 2E).

### Negative Regulation of TBK1_tv3 on IFN and ISGs Mediated by RLR Pathway

**FIGURE 3:**
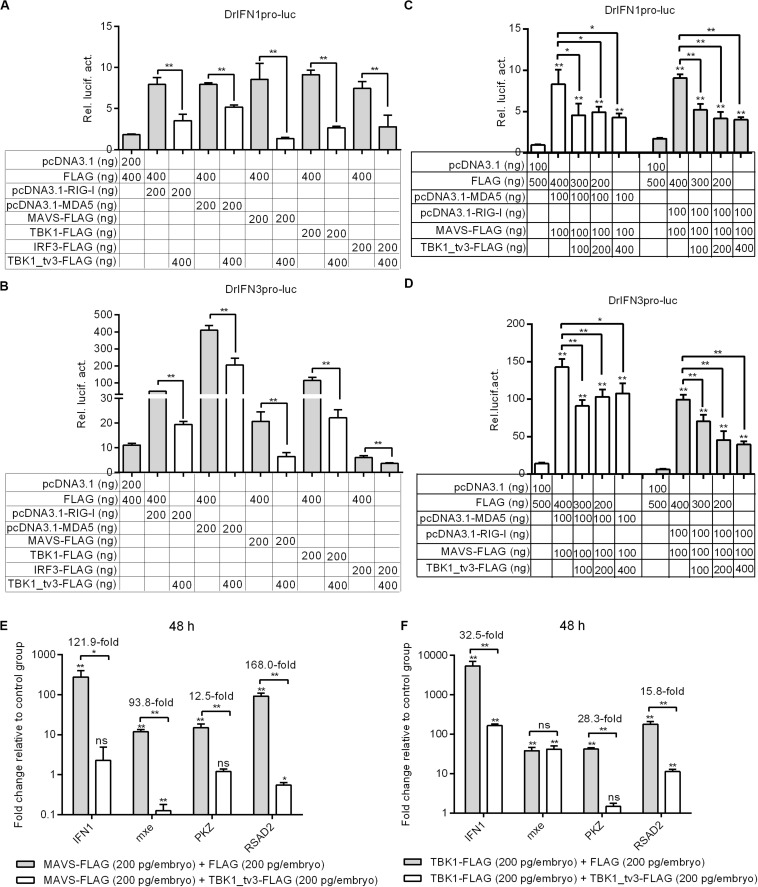
TBK1_tv3 negatively regulates type I IFN mediated by RLR signaling pathway. **(A)** Effect of zebrafish TBK1_tv3 on the IFN1 promoter activity mediated by RLR signaling pathway. **(B)** Effect of zebrafish TBK1_tv3 on the IFN3 promoter activity mediated by RLR signaling pathway. **(C)** Effect of zebrafish TBK1_tv3 on the IFN1 promoter activity mediated by MDA5 or RIG-I and MAVS. **(D)** Effect of zebrafish TBK1_tv3 on the IFN3 promoter activity mediated by MDA5 or RIG-I and MAVS. For **A–D**, EPC cells seeded overnight in 24-well plates at 3 × 10^5^ cells per well were transiently transfected with various indicated plasmids with indicated DNA concentration, together with 25 ng Renilla and 250 ng IFN1 or IFN3 reporter plasmids. After 48 h post-transfection, the cells were harvested for the detection of luciferase activity. **(E)** Effects of zebrafish TBK1_tv3 on expression of IFN and IFN-stimulated genes induced by MAVS. **(F)** Effects of zebrafish TBK1_tv3 on expression of IFN and IFN-stimulated genes induced by TBK1. For **E,F**, zebrafish embryos were microinjected at the one-cell stage with 100 ng of indicated expression construct. At 48 h post-fertilization, 50 embryos for each sample were used for RNA extraction. The p3xFLAG-injected group was used for control. Data represented means ± SEM (*n* = 3) and were tested for statistical significance using one-way ANOVA followed by Tukey test. *p < 0.05; **p < 0.01; and ns, not significant. The asterisk above the error bars indicated statistical significance using the group transfected with empty plasmid as the control group. The asterisk above the bracket indicated statistical significance between the two groups connected by the bracket.

Because piscine RLR pathway is a pivotal factor in the activation of IFN production (23), the function of TBK1_tv3 in RLR-mediated IFN production was characterized. Zebrafish IFN1 activity was activated by those key genes involved in RLR pathway, whereas the induction was impaired in the existence of TBK1_tv3 (Figure 3A). Similar results were observed for zebrafish IFN3 activity (Figure 3B).

A previous study showed that the antiviral gene NOD2 acting as a negative regulator of RIG-I signaling seemed likely due to the consequence of the CARDs of RIG-I away from MAVS (24). To rule out the possibility that the negative regulations of zebrafish TBK1_tv3 on the RLRs and MAVS were due to the consequence of the CARDs of RLRs away from MAVS, EPC cells were transfected with both RLR receptor such as MDA5 or RIG-I and the adaptor protein MAVS. Similarly, the overexpression of TBK1_tv3 reduced the activation of zebrafish IFN1 and IFN3 promoters induced by MDA5 or RIG-I and MAVS normal forms (Figures 3C,D).

MAVS and TBK1 are the essential adaptor protein and kinase in RLR antiviral innate immune signaling, respectively. The overexpression of piscine MAVS and TBK1 resulted in significant accumulation of IFNs and ISGs, which correlated with a marked enhancement of protection against SVCV infection (18, 21). A recent study has shown that zebrafish TBK1_tv1 and TBK1_tv2 negatively regulated MAVS- and TBK1-mediated innate immunity (18). In the present work, the transcriptional levels of IFN1 and ISGs were analyzed to establish whether zebrafish TBK1_tv3 was also involved in the negative regulation of MAVS- and TBK1-mediated innate immunity. Compared with MAVS and FLAG vector treatment, the expression of IFN1 in the group microinjected with MAVS and TBK1_tv3 decreased more than 120-fold at 48 h. TBK1_tv3 also significantly inhibited the production of mxe (93.8-fold), PKZ (12.5-fold), and RSAD2 (168.0-fold) induced by MAVS (Figure 3E). Compared with TBK1 and FLAG vector treatment, TBK1_tv3 significantly inhibited the production of IFN1 (32.5-fold), PKZ (28.3-fold), and RSAD2 (15.8-fold) induced by TBK1 (Figure 3F).

### TBK1_tv3 Interacts With TBK1 and IRF3 to Prevent Them From Binding With Each Other and Themselves

**FIGURE 4:**
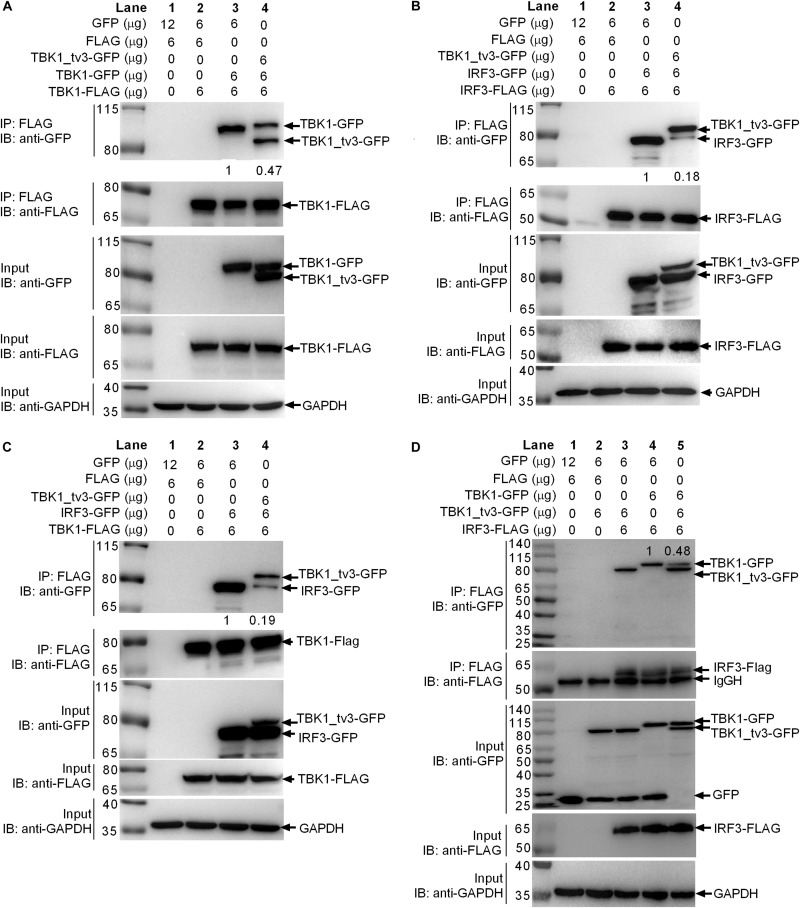
TBK1_tv3 interacts with TBK1 and IRF3 to disrupt the formation of TBK1 homodimers, IRF3 homodimers, and TBK1–IRF3 complex. **(A)** TBK1_tv3 inhibited the formation of TBK1 homodimers. **(B)** TBK1_tv3 inhibited the formation of IRF3 homodimers. **(C)** TBK1_tv3 interacted with TBK1 to inhibit the formation of TBK1–IRF3 complex. **(D)** TBK1_tv3 interacted with IRF3 to inhibit the formation of TBK1–IRF3 complex. For **A–D**, co-IP was performed with anti-FLAG-conjugated agarose beads in EPC cells. The cell lysates and bound proteins were analyzed by immunoblotting with the indicated Abs. The expression ratio was quantified by Quantity One. All experiments were repeated for at least three times with similar results.

Because the KD, ULD, and SDD (CCD1) of TBK1 are involved in the dimer formation, it will be of interest to determine whether TBK1_tv3 isoform including all these three domains might interact with TBK1 normal form to perturb TBK1 homo-dimerization. To investigate this, anti-FLAG-conjugated agarose beads were used to precipitate TBK1-FLAG-containing protein complex from EPC cells and found TBK1-GFP (lane 3 in Figure 4A) or TBK1-GFP/TBK1_tv3-GFP (lane 4 in Figure 4A) in this complex. Compared with TBK1-GFP band in lane 3, a significantly decreased (TBK1-FLAG)–(TBK1-GFP) interaction was observed (lanes 3 and 4 in Figure 4A). Thus, TBK1_tv3 was interacted with TBK1 to interfere TBK1 dimerization when overexpressed in EPC cells.

Activated TBK1 could phosphorylate IRF3 and trigger IRF3 dimerization. Whether the interaction between TBK1_tv3 and IRF3 might impede IRF3 homo-dimerization was further investigated. Results from a similar co-immunoprecipitation experiment also showed that TBK1_tv3 was capable of impeding the formation of IRF3 dimerization when overexpressed in EPC cells (Figure 4B).

The formation of functional TBK1–IRF3 complex is critical for IRF3 phosphorylation and type I IFN induction. Next, we interrogated whether the interaction between TBK1_tv3 and TBK1 or IRF3 might impede the formation of TBK1–IRF3 complex. Anti-FLAG-conjugated agarose beads were used to precipitate TBK1-FLAG-containing protein complex, and found IRF3-GFP (lane 3 in Figure 4C) or TBK1_tv3-GFP/IRF3-GFP (lane 4 in Figure 4C) in this complex. Compared with IRF3-GFP band in lane 3, a significantly decreased TBK1–IRF3 interaction was observed (lanes 3 and 4 in Figure 4C). Similarly, anti-FLAG-conjugated agarose beads were used to precipitate IRF3-FLAG-containing protein complex, and found TBK1_tv3-GFP (lanes 3 and 5 in Figure 4D) and TBK1-GFP (lanes 4 and 5 in Figure 4D) in this complex. Compared with TBK1-GFP band in lane 4, a significantly decreased IRF3–TBK1 interaction was observed (lanes 4 and 5 in Figure 4D). Therefore, TBK1_tv3 was interacted with TBK1 and IRF3 to interfere the formation of TBK1–IRF3 complex when overexpressed in EPC cells.

### TBK1_tv3 Promotes the Degradation of TBK1 and IRF3 Through Different Protein Degradation Pathways

**FIGURE 5:**
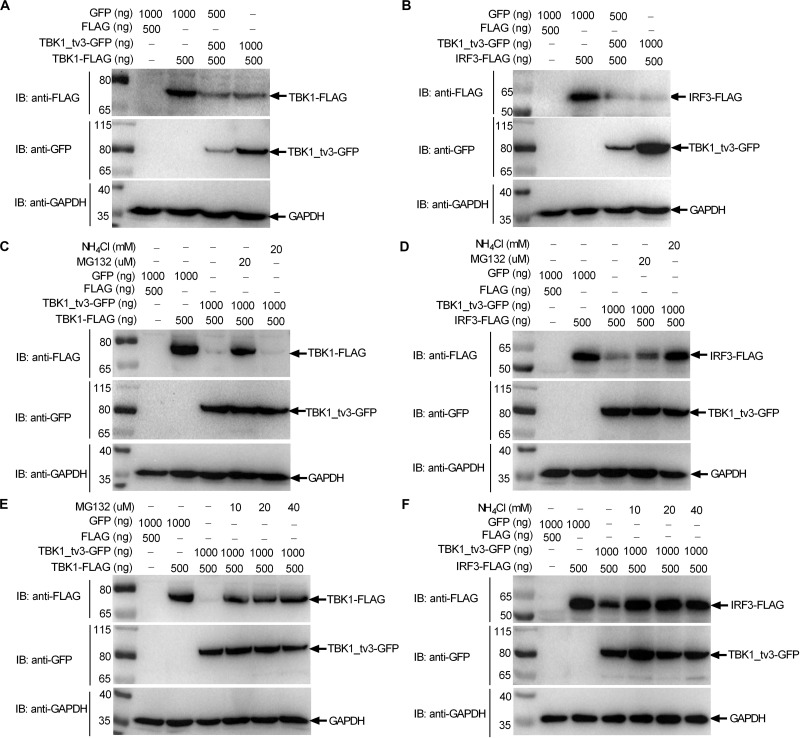
TBK1_tv3 promotes proteasomal degradation of TBK1 and lysosomal degradation of IRF3. **(A)** The effect of TBK1_tv3 on the protein expression of TBK1. **(B)** The effect of TBK1_tv3 on the protein expression of IRF3. For **A,B**, EPC cells seeded in six-well plates were transfected with various indicated plasmids with indicated DNA concentration. After 48 h post-transfection, cell lysates were analyzed by immunoblotting using the indicated Abs. **(C)** TBK1_tv3 promotes the degradation of TBK1 by ubiquitination pathway. **(D)** TBK1_tv3 induced the degradation of IRF3 by lysosome pathway. **(E)** TBK1_tv3 promotes the degradation of TBK1 by ubiquitination pathway independent of the dose variation. **(F)** TBK1_tv3 induced the degradation of IRF3 by lysosome pathway independent of the dose variation. For **C–F**, EPC cells seeded in six-well plates were transfected with various indicated plasmids with indicated DNA concentration. After 48 h post-transfection, cells were treated with DMSO, MG132, or NH_4_Cl with indicated concentration for 6 h or left untreated. Following this, cell lysates were analyzed by immunoblotting using the indicated Abs. All experiments were repeated for at least three times with similar results.

Protein degradation is the main strategy used to modulate protein function in biological processes both by host and pathogens. Because TBK1_tv3 interferes with TBK1/IRF3 homo- and hetero-dimerization, we then examined whether TBK1_tv3 might destabilize TBK1 and IRF3 proteins. WB analysis with tag-specific Abs showed that with the addition of TBK1_tv3, the amount of TBK1 and IRF3 was significantly decreased (Figures 5A,B). To delineate the mechanisms involved in TBK1_tv3 degrading with TBK1 and IRF3 expression during the posttranscriptional process, the effects of MG132 (inhibitor for the proteasome) and NH_4_Cl (the lysosomal inhibitor) on TBK1_tv3-mediated degradation of TBK1 and IRF3 were investigated in EPC cells. The TBK1_tv3-mediated degradation of TBK1 was partly inhibited by 20 μM MG132, but not for 20 mM NH_4_Cl (Figure 5C). However, the TBK1_tv3-mediated degradation of IRF3 was completely inhibited by 20 mM NH_4_Cl, but not for 20 μM MG132 (Figure 5D). The inhibition of TBK1_tv3-mediated degradation of TBK1 by MG132 and the inhibition of TBK1_tv3-mediated degradation of IRF3 by NH_4_Cl were independent of the dose variation (Figures 5E,F). Similar results were obtained in HEK 293T cells (data not shown). These data demonstrate that TBK1 is degraded by TBK1_tv3 through the proteasome pathway, and IRF3 is degraded by TBK1_tv3 through the lysosomal pathway.

### TBK1_tv3 Promotes TBK1 Degradation via K48-Linked Ubiquitination at the K251,K256, and K271 Sites

**FIGURE 6:**
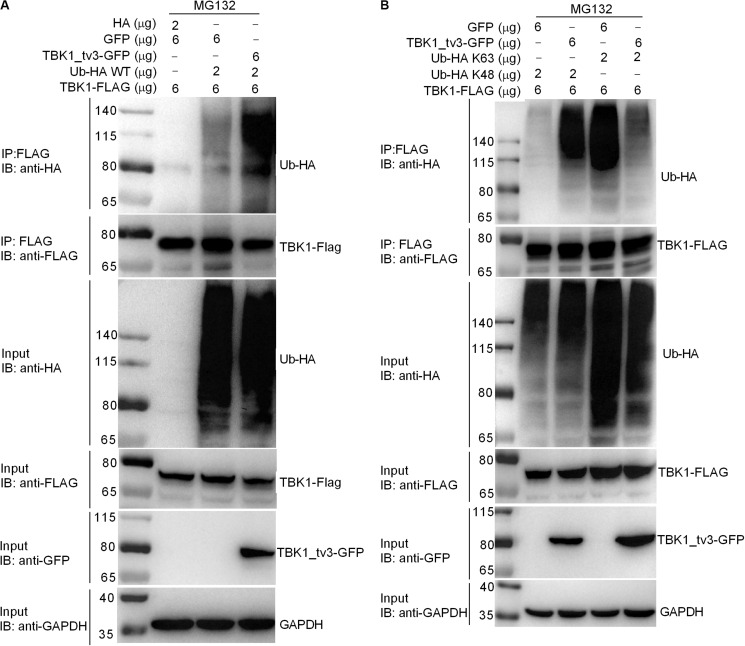
TBK1_tv3 promotes K48-linked ubiquitination of TBK1. **(A)** TBK1_tv3 promotes the ubiquitination of TBK1. HEK 293T cells were seeded in 10-cm^2^ dishes and transfected with 6 μg TBK1-FLAG, 6 μg TBK1_tv3-GFP, empty vector, and 2 μg Ub-HA WT. At 18 h post-transfection, the cells were treated with MG132 for 6 h. Cell lysates were IP with anti-FLAG-conjugated agarose beads. The cell lysates and bound proteins were analyzed by immunoblotting with the indicated Abs. **(B)** TBK1_tv3 promotes the K48-linked ubiquitination of TBK1. HEK 293T cells were seeded in 10-cm^2^ dishes and transfected with 6 μg TBK1-FLAG, 6 μg TBK1_tv3-GFP, empty vector, 2 μg Ub-HA K48, or 2 μg Ub-HA K63. At 18 h post-transfection, the cells were treated with MG132 for 6 h. Cell lysates were IP with anti-FLAG-conjugated agarose beads. The cell lysates and bound proteins were analyzed by immunoblotting with the indicated Abs. All experiments were repeated for at least three times with similar results.

We then determined whether TBK1_tv3 elicited the ubiquitination of TBK1. When ectopically expressed TBK1_tv3 was immunoprecipitated by FLAG, the ubiquitination of TBK1 was obviously increased in the presence of TBK1_tv3 (Figure 6A). It has been documented that the accumulation and function of TBK1 is regulated by both K48-linked and K63-linked ubiquitination (8, 9). To determine the specific ubiquitination type of TBK1, we transfected HEK 293T cells with FLAG-TBK1, TBK1_tv3-GFP, HA-tagged K48-linked ubiquitin (Ub-HA K48), or K63-linked ubiquitin (Ub-HA K63). Immunoprecipitation and immunoblot analyses revealed that TBK1_tv3 promoted more K48-linked ubiquitination of TBK1 whereas it attenuated the K63-linked ubiquitination of TBK1 (Figure 6B).

**FIGURE 7:**
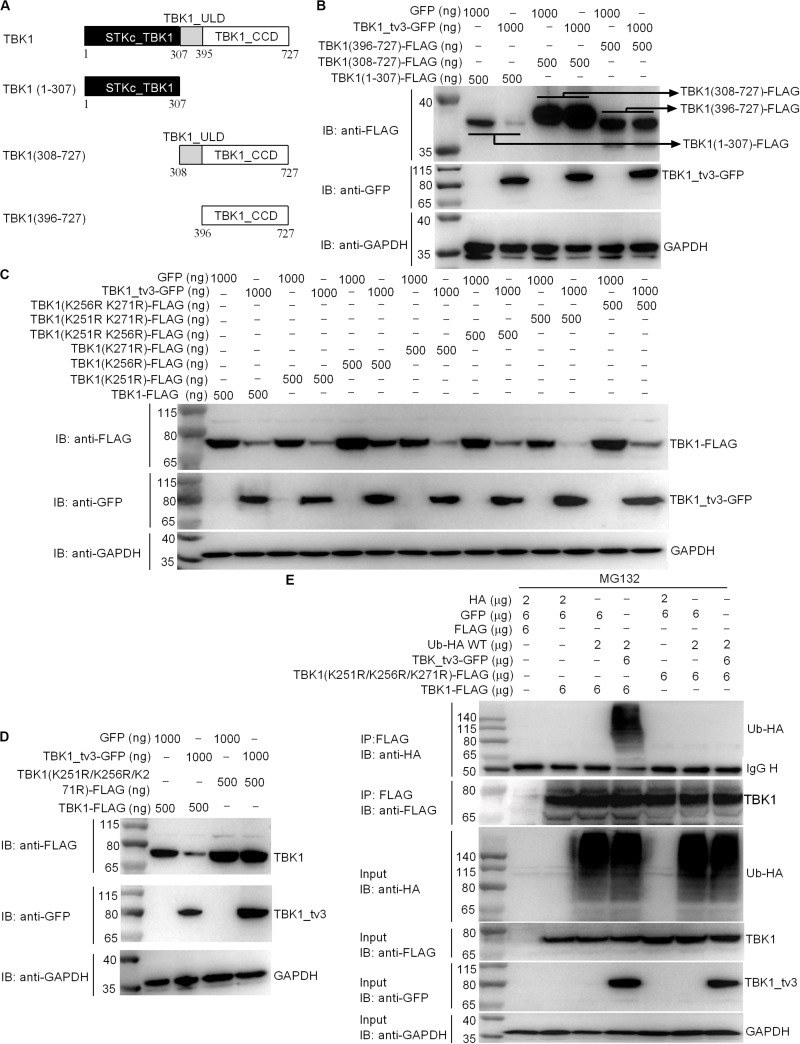
The K251, K256, and K271 sites are vital for K48-linked ubiquitination of TBK1. **(A)** A schematic of TBK1 and truncated mutants. **(B)** The effect of TBK1_tv3 on the protein expression of TBK1 truncated mutants. **(C,D)** The effects of TBK1_tv3 on the protein expression of TBK1 with site mutants. For **B–D**, EPC cells were seeded in six-well plates and transfected with various indicated plasmids with indicated DNA concentration. After 48 h post-transfection, cell lysates were analyzed by immunoblotting using the indicated Abs. **(E)** The effect of TBK1_tv3 on the K48-linked ubiquitination of wild type and TBK1 with site mutant (K251R, K256R, and K271R). HEK 293T cells were seeded in 10-cm^2^ dishes and transfected with 2 μg HA, 6 μg p3xFLAG, 6 μg pTurboGFP, 2 μg Ub-HA WT, 6 μg TBK1_tv3-GFP, 6 μg TBK1 (K251R, K256R, and K271R)-FLAG, and 6 μg TBK1-FLAG with the indicated combination. At 18 h post-transfection, the cells were treated with MG132 for 6 h. Cell lysates were IP with anti-FLAG-conjugated agarose beads. The cell lysates and bound proteins were analyzed by immunoblotting with the indicated Abs. All experiments were repeated for at least three times with similar results.

We further attempted to identify the specific domain(s) and site(s) of TBK1_tv3-mediated ubiquitination in TBK1. Among three TBK1 truncations examined (Figure 7A), the fragment containing ULD and/or CCD domain(s) did not show the enhanced degradation upon TBK1_tv3 overexpression, but the STKc_TBK1 (namely KD) domain did (Figure 7B), suggesting that the KD of TBK1 may contain sites for TBK1_tv3-mediated ubiquitination.

We identified three potential ubiquitination sites (K251, K256, and K271) in the KD, and substituted them with arginine (R) to create three single-site mutants (K251R, K256R, and K271R), three double-site mutants (K251R/K256R, K251R/K271R, and K256R/K271R), and one three-site mutant (K251R/K256R/K271R). Compared with wild-type TBK1, only the three-site mutant (K251R/K256R/K271R) completely abolished TBK1_tv3-mediated degradation of TBK1 (Figures 7C,D). Furthermore, TBK1_tv3-mediated ubiquitination of TBK1 was completely abolished by the K251R/K256R/K271R three-site mutant (Figure 7E).

## Discussion

Alternative splicing is an important regulatory mechanism in innate immunity and splice isoforms of RLR pathway including RIG-I, MDA5, LGP2, MAVS, STING, TBK1, IRF3, IFNs, and IFN receptors have been described (16). In mammals, TBK1 isoform (TBK1s) generated by alternative splicing was reported to inhibit RIG-I- but not MAVS- or TBK1-mediated activation of IFN promoter, and disrupt the interaction of RIG-I with MAVS (17). In teleost fish, our previous study showed that zebrafish TBK1_tv1 and TBK1_tv2 isoforms inhibited RIG-I-, MAVS-, TBK1-, and IRF3-mediated activation of IFN promoters in response to SVCV infection, disrupted the formation of a functional TBK1–IRF3 complex, and impeded the phosphorylation of IRF3 mediated by TBK1 (18). In this study, we identified and characterized zebrafish TBK1_tv3 as a novel transcript isoform that inhibited type I IFN production by promoting TBK1 and IRF3 degradation through different protein degradation pathways. The present study firstly reveals that TBK1 isoform not only mediates K48-linked ubiquitination at Lys251, Lys256, and Lys271 sites of TBK1 to promote TBK1 proteasomal degradation but it also might function as a deubiquitinase to disrupt K63-linked polyubiquitination of TBK1. Our findings provide new mechanistic insight on the regulation of TBK1 and IRF3 activities by TBK1 isoform, which are different from previous reports (17, 18).

Although mammalian and piscine TBK1 isoforms are generated by exon skipping, the reported TBK1 isoforms contain the different domain composition. Mouse TBK1s lacking exons 3–6 results in translation from the second ATG and an in-frame deletion of the KD (17). Zebrafish TBK1_tv1 lacking exons 3–4 and TBK1_tv2 lacking exons 4–18 are generated by the usage of the same ATG transcription start site and stop codon (18). Zebrafish TBK1_tv1 isoform consists of an incomplete KD, ULD, CCD1, and CCD2. However, TBK1_tv2 only contains an incomplete KD and CCD2, and lacks ULD and CCD1 domains. Different from zebrafish TBK1_tv1 and TBK1_tv2, TBK1_tv3 is generated by more complicated exon skipping with the usage of the same ATG transcription start site and different stop codon site when compared with TBK1 normal form. Zebrafish TBK1_tv3 reserves the intact KD and ULD domains, and contains incomplete CCD domains (Figure 1C). In mammals, the KD of TBK1 is responsible for kinase activity, the ULD for the control of kinase activation, substrate presentation and downstream signaling pathways, CCD1 domain for TBK1 dimerization, and CCD2 domain for adaptor binding and TBK1 activation (25–28). The different domain composition of TBK1 isoforms may lead to different regulation mechanisms of TBK1 isoforms in the antiviral innate immunity.

Excessive production of IFNs has been suggested to be destructive rather than protective. In teleost fish, several proteins have been demonstrated to contribute to the negative regulation of IFN response. First, grass carp SARM1 inhibited GCRV-triggered IFN-I response by affecting the expressions of TRIF, MyD88, IPS-1, TRAF6, TBK1, IRF3, and IRF7 in TRIF-, MyD88-, and IPS-1-dependent pathways (29). In addition, zebrafish STAT6, FGFR3, MVP, NDRG1a, RPZ5, and FTRCA1 suppressed IFN production by attenuating the kinase activity of TBK1 (30, 31), degrading TBK1 (32, 33) and phosphorylated IRF7 (34, 35), or attenuating IRF7 transcription (36). Using the luciferase reporter assay system, our previous study (18) and the present study revealed that TBK1 isoforms, TBK1_tv1–TBK1_tv3, had the same function in inhibiting RLR-mediated type I IFN production with or without SVCV infection. In addition, TBK1 isoforms significantly decreased the mRNA levels of IFN1 and ISGs in zebrafish embryos induced by the key adaptor protein MAVS or TBK1 kinase of RLR pathway. In mammals, previous studies have demonstrated that CCD2 domain of TBK1 is responsible for adaptor binding and TBK1 activation, and that IRF3 is targeted for degradation to turn off IFN production (26, 37, 38). In the present study, zebrafish TBK1_tv3 lacking CCD2 domain failed to induce the expression of IFN1 and ISGs, which could be due to the deactivation of TBK1 activity and the degradations of TBK1 and IRF3 mediated by zebrafish TBK1_tv3. Hence, all these results clearly demonstrate that TBK1 isoforms are negative regulators of RLR-mediated type I IFN production.

The formation of functional TBK1-containing complexes including TBK1, IKKε, TRAF3, IRF3, and other adaptors (TRIF, MAVS, and STING) is necessary for IRF3 phosphorylation and its nuclear translocation, which leads to the production of type I IFN in antiviral immune responses (8). Therefore, preventing the formation of functional TBK1-containing complexes is a major mechanism for negative regulation of IFN response. However, in teleost fish, only zebrafish TBK1_tv1 and TBK1_tv2 isoforms were found to impede the formation of functional TBK1–IRF3 complex (18). In this study, zebrafish TBK1_tv3 is also identified as a negative regulator that inhibits RLR-mediated IFN production by impeding the formation of TBK1–TBK1 (TBK1 dimerization), TBK1–IRF3 complex and IRF3 dimerization.

The ubiquitin–proteasome, lysosomal, and autophagosome pathways are the main pathways of protein degradation (39). As a pivotal kinase of RLR antiviral immunity, the activity of TBK1 must be tightly regulated to maintain immune homeostasis (8). Mammalian E3 ligases such as TRIP can specifically target K48-linked ubiquitination of TBK1 to promote the degradation of TBK1 through proteasome pathway and terminate downstream signaling transduction (40). However, several deubiquitinases such as CYLD disrupt K63-linked polyubiquitination to terminate TBK1-mediated signaling transduction (41). Recently, zebrafish MVP and FTRCA1 were found to mediate the lysosome-dependent degradation of TBK1 (32, 33). Here, we show that zebrafish TBK1_tv3 is a key kinase that mediates K48-linked ubiquitination at Lys251, Lys256, and Lys271 of TBK1 and promotes TBK1 proteasomal degradation, thus functioning as a negative regulator of type I IFN production. Interestingly, the present study also showed that zebrafish TBK1_tv3 might function as a deubiquitinase which could disrupt K63-linked polyubiquitination of TBK1.

IRF3 is directly implicated in the transcriptional induction of type I IFNs, and IRF3 activity is controlled by posttranscriptional modifications including phosphorylation, ubiquitination, and deubiquitination. RAUL, FoxO1, TRIM26, and c-Cbl are E3 ligases that induce K48-linked ubiquitination and degradation of IRF3 (37, 38, 42, 43). The deubiquitinase OTUD1 negatively regulates type I IFN induction by atypical K6-linked ubiquitination and deubiquitination process of IRF3 (44). Previously, it has been reported that the overexpression of piscine TBK1 induced IRF3 phosphorylation, whereas the overexpression of TBK1_tv1 and TBK1_tv2 had little effect on IRF3 phosphorylation (18). However, none of the reports was involved in TBK1 isoform-mediated degradation of IRF3. In this study, we confirmed that IRF3 could interact with zebrafish TBK1_tv3. Interestingly, zebrafish TBK1_tv3-mediated degradation of IRF3 was blocked by the lysosome inhibitor NH_4_Cl but not the proteasome inhibitor MG132. These results indicate that zebrafish TBK1_tv3 regulates stability of IRF3 protein in a lysosomal-dependent manner. However, the exact mechanism on how zebrafish TBK1_tv3 mediates lysosomal-dependent degradation of IRF3 is currently unknown, which needs to be further studied.

**FIGURE 8:**
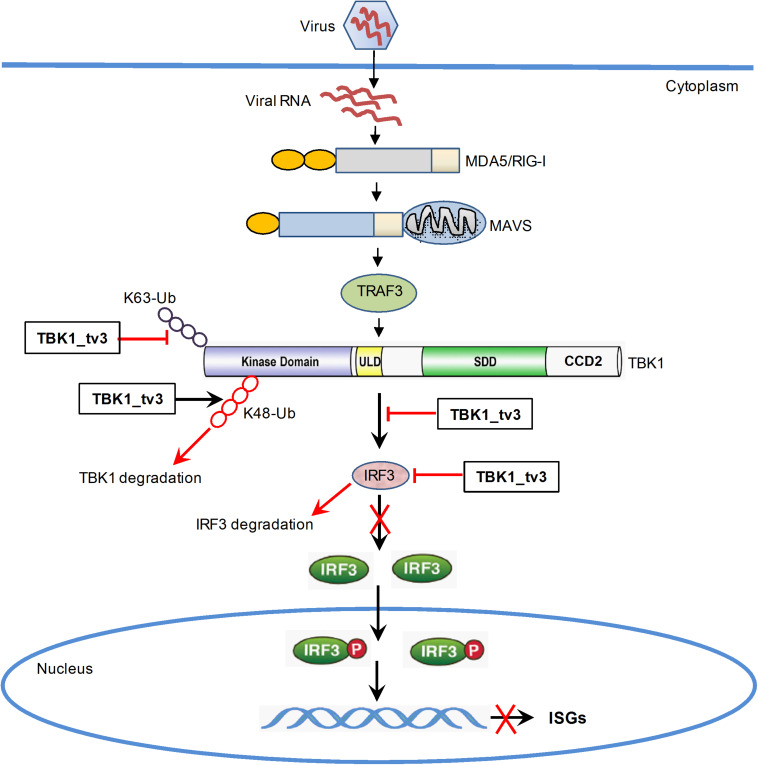
Proposed model illustrating the negative regulation of TBK1_tv3 in the homeostasis of innate immune signaling.

In summary, our study has identified the essential role of TBK1_tv3 isoform in negatively regulating type I IFN signaling and antiviral immunity *in vitro* and *in vivo*. TBK1_tv3 acts as a negative regulator in RLR pathway by disrupting TBK1/IRF3 homo- and hetero-dimerization. Mechanistically, TBK1_tv3 targets TBK1 normal form, and functions as an E3 ligase to induce K48-linked ubiquitination at Lys251, Lys256, and Lys271 residues of TBK1 and a deubiquitinase to disrupt K63-linked polyubiquitination of TBK1, which consequently leads to proteasomal degradation of TBK1. TBK1_tv3 also targets IRF3 for protein degradation in a lysosomal manner (Figure 8). These findings provide new insights into a previously unrecognized role for TBK1 isoform in the homeostasis of innate immune signaling and molecular mechanisms by which TBK1_tv3 targets activated TBK1 and IRF3 for ubiquitination and degradation.

## Data Availability Statement

The original contributions presented in the study are included in the article/Supplementary Material, further inquiries can be directed to the corresponding author/s.

## Ethics Statement

The animal study was reviewed and approved by Institute of Hydrobiology, Chinese Academy of Sciences (Approval ID: IHB 2013724).

## Author Contributions

MC conceived and designed the experiments. JZ, XW, and YH performed the experiments and analyzed the data. MC and JZ wrote the article. MC revised the article. All authors reviewed the article.

## Conflict of Interest

The authors declare that the research was conducted in the absence of any commercial or financial relationships that could be construed as a potential conflict of interest.
