# Supplementary material for: A Novel Transcript Isoform of TBK1 Negatively Regulates Type I IFN Production by Promoting Proteasomal Degradation of TBK1 and Lysosomal Degradation of IRF3
Source: Front Immunol. 2020 Sep 30;11:580864. doi: 10.3389/fimmu.2020.580864 (PMC7554342; doi:10.3389/fimmu.2020.580864)
Supplement: Supplementary file 1 [file Table_1.DOCX]

Table S1. Primer information

| Name | Sequence | Application |
| --- | --- | --- |
| TBK1F | GTCAAGCTTTCTACAGTCATCATGCAGAGT | Ligated to p3xFLAG-CMV™-14 vector or GFP vector |
| TBK1R | GAAGGTACCATCCGCTCCACTGTCCTCA |  |
| TBK1_tv3F | GTCAAGCTTTCTACAGTCATCATGCAGAGT |  |
| TBK1_tv3R | GTTGGTACCCCTTCAGTTTCTTCATCCCA |  |
| IRF3F | GAGAAGCTTACCATGACTCAAGCAAAACC |  |
| IRF3R | GAAGGTACCCAGCAGAGCTCCATCATTTG |  |
| pcDNA3.1-RIG-IF | CGCGGATCCACCATGGCGTACGAGCTGGAGAAGGAGA | Ligated to pcDNA3.1 vector |
| pcDNA3.1-RIG-IF | CGGGGTACCGTTGACCAGCGCCCATG |  |
| pcDNA3.1-MDA5F | GACCTCGAGCGATGGATCCAAACATGAGCAG |  |
| pcDNA3.1-MDA5R | CGGGGTACCGTTAGTGTCCATATCTTCATC |  |
| p3×FLAG-MAVSF | CGGGGTACCAATGGCTTCACTGACACGTG | Ligated to p3xFLAG-CMV™-14 vector |
| p3×FLAG-MAVSR | CACGGATCCATGATTGAGCTTCCAGGC |  |
| TBK1_tv3F | TGGGAACACACCCCGATGGCGTCT | Quantitative real-time PCR or RT-PCR |
| TBK1_tv3R | CCAGCTCCTTCACCCTTCAGTTTCTT |  |
| IFN1F | GTCAGGACTAAAAACTTCAC |  |
| IFN1R | TCTTAATACACGCAAAGATGAGAACT |  |
| mxeF | TGAAGATGGCATCCACAGTT |  |
| mxeR | TCTTTCTGCAAGCAGGGGT |  |
| PKZF | GGAGCACCGTACAGGACATT |  |
| PKZR | CTCGGGCTTTATTTGCTCTG |  |
| RSAD2F | AGCAGATCACCGCTCTCAAT |  |
| RSAD2R | CCAGACACTGGATGCTCTGA |  |
| SVCV-PF | TGAGGAGGAATGGGAATCAG |  |
| SVCV-PF | AGCTGACTGTCGGGAGATGT |  |
| SVCV-NF | GCCGATTATCCTTCCACCTT |  |
| SVCV-NF | TCACTTGCCCTTCCCACTCT |  |
| SVCV-GF | CGACCTGGATTAGACTTG |  |
| SVCV-GF | AATGTTCCGTTTCTCACT |  |
